# Supplementary material for: Overlapping cell population expression profiling and regulatory inference in C. elegans
Source: BMC Genomics. 2016 Feb 29;17:159. doi: 10.1186/s12864-016-2482-z (PMC4772325; doi:10.1186/s12864-016-2482-z)
Supplement: Additional file 13: — Web supplement. (DOC 21 kb) [file 12864_2016_2482_MOESM13_ESM.zip › sortWeb/clusters/hier.300.clusters/51.html]

Cluster 51 

## Cluster 51

### Expression

| cnd-1 rep. 1 | cnd-1 rep. 2 | cnd-1 rep. 3 | pha-4 rep. 1 | pha-4 rep. 2 | pha-4 rep. 3 | ceh-27 | ceh-36 | ceh-6 | F21D5.9 | mir-57 | mls-2 | pal-1 | pros-1 | ttx-3 | unc-130 | hlh-16 | irx-1 | ceh-6 (+) hlh-16 (+) | ceh-6 (+) hlh-16 (-) | ceh-6 (-) hlh-16 (+) | cnd-1 singlets | pha-4 singlets | 0 | 60 | 120 | 150 | 180 | 240 | 330 | 390 | 420 | 480 | 540 | 570 | 600 | 630 | 660 | NAME | Functional description |
| --- | --- | --- | --- | --- | --- | --- | --- | --- | --- | --- | --- | --- | --- | --- | --- | --- | --- | --- | --- | --- | --- | --- | --- | --- | --- | --- | --- | --- | --- | --- | --- | --- | --- | --- | --- | --- | --- | --- | --- |
|  |  |  |  |  |  |  |  |  |  |  |  |  |  |  |  |  |  |  |  |  |  |  |  |  |  |  |  |  |  |  |  |  |  |  |  |  |  | H06H21.11 |  |
|  |  |  |  |  |  |  |  |  |  |  |  |  |  |  |  |  |  |  |  |  |  |  |  |  |  |  |  |  |  |  |  |  |  |  |  |  |  | F13E6.5 |  |
|  |  |  |  |  |  |  |  |  |  |  |  |  |  |  |  |  |  |  |  |  |  |  |  |  |  |  |  |  |  |  |  |  |  |  |  |  |  | *ostd-1* | OligoSaccharylTransferase, Delta subunit |
|  |  |  |  |  |  |  |  |  |  |  |  |  |  |  |  |  |  |  |  |  |  |  |  |  |  |  |  |  |  |  |  |  |  |  |  |  |  | *tag-189* | Temporarily Assigned Gene name |
|  |  |  |  |  |  |  |  |  |  |  |  |  |  |  |  |  |  |  |  |  |  |  |  |  |  |  |  |  |  |  |  |  |  |  |  |  |  | B0205.5 |  |
|  |  |  |  |  |  |  |  |  |  |  |  |  |  |  |  |  |  |  |  |  |  |  |  |  |  |  |  |  |  |  |  |  |  |  |  |  |  | F38E1.9 |  |
|  |  |  |  |  |  |  |  |  |  |  |  |  |  |  |  |  |  |  |  |  |  |  |  |  |  |  |  |  |  |  |  |  |  |  |  |  |  | *irld-65* | Insulin/EGF-Receptor L Domain protein |
|  |  |  |  |  |  |  |  |  |  |  |  |  |  |  |  |  |  |  |  |  |  |  |  |  |  |  |  |  |  |  |  |  |  |  |  |  |  | F37B12.3 |  |
|  |  |  |  |  |  |  |  |  |  |  |  |  |  |  |  |  |  |  |  |  |  |  |  |  |  |  |  |  |  |  |  |  |  |  |  |  |  | C16C2.5 |  |
|  |  |  |  |  |  |  |  |  |  |  |  |  |  |  |  |  |  |  |  |  |  |  |  |  |  |  |  |  |  |  |  |  |  |  |  |  |  | R186.3 |  |
|  |  |  |  |  |  |  |  |  |  |  |  |  |  |  |  |  |  |  |  |  |  |  |  |  |  |  |  |  |  |  |  |  |  |  |  |  |  | C27F2.9 |  |
|  |  |  |  |  |  |  |  |  |  |  |  |  |  |  |  |  |  |  |  |  |  |  |  |  |  |  |  |  |  |  |  |  |  |  |  |  |  | B0035.3 |  |
|  |  |  |  |  |  |  |  |  |  |  |  |  |  |  |  |  |  |  |  |  |  |  |  |  |  |  |  |  |  |  |  |  |  |  |  |  |  | C43E11.12 |  |
|  |  |  |  |  |  |  |  |  |  |  |  |  |  |  |  |  |  |  |  |  |  |  |  |  |  |  |  |  |  |  |  |  |  |  |  |  |  | Y39E4B.5 |  |
|  |  |  |  |  |  |  |  |  |  |  |  |  |  |  |  |  |  |  |  |  |  |  |  |  |  |  |  |  |  |  |  |  |  |  |  |  |  | Y55F3AR.2 |  |
|  |  |  |  |  |  |  |  |  |  |  |  |  |  |  |  |  |  |  |  |  |  |  |  |  |  |  |  |  |  |  |  |  |  |  |  |  |  | R06C1.4 |  |
|  |  |  |  |  |  |  |  |  |  |  |  |  |  |  |  |  |  |  |  |  |  |  |  |  |  |  |  |  |  |  |  |  |  |  |  |  |  | K08D12.3 |  |
|  |  |  |  |  |  |  |  |  |  |  |  |  |  |  |  |  |  |  |  |  |  |  |  |  |  |  |  |  |  |  |  |  |  |  |  |  |  | *her-1* | HERmaphrodization of XO animals |
|  |  |  |  |  |  |  |  |  |  |  |  |  |  |  |  |  |  |  |  |  |  |  |  |  |  |  |  |  |  |  |  |  |  |  |  |  |  | T28B11.22 |  |
|  |  |  |  |  |  |  |  |  |  |  |  |  |  |  |  |  |  |  |  |  |  |  |  |  |  |  |  |  |  |  |  |  |  |  |  |  |  | T14E8.6 |  |

### Phenotypes enriched

none found

### Anatomy terms enriched

none found

### GO terms enriched

none found

### Expression clusters enriched

none found

### Motifs enriched

|  |  |  |  |  |  |
| --- | --- | --- | --- | --- | --- |
| **Motif** | **Logo** | **Possible orthologs** | **Number of motifs in cluster** | **Enrichment** | **FDR corrected p** |
| pTH5260 |  | lin-22 | 13 | 2.98 | 0.0052 |
| pTH1294 |  | mel-28 | 13 | 2.84 | 0.0078 |
| V$FREAC3\_01 |  | let-381 | 13 | 2.37 | 0.0360 |
| Mitf\_SANGER\_5\_FBgn0263112 |  | hlh-30 | 12 | 2.54 | 0.0370 |
| pTH9242 |  | mel-28 | 11 | 2.76 | 0.0380 |
| NR2F6\_1 |  | nhr-2 | 8 | 3.70 | 0.0450 |
| PBX1\_do |  | ceh-20 | 16 | 1.88 | 0.0470 |
| ems\_FlyReg\_FBgn0000576 |  | ceh-2 | 14 | 2.12 | 0.0490 |

### Correlated (and anti-correlated) transcription factors

|  |  |
| --- | --- |
| **Transcription factor** | **Correlation** |
| ceh-53 | 0.59 |
| nhr-122 | 0.58 |
| snu-23 | 0.53 |
| D2030.7 | 0.53 |
| nhr-207 | 0.52 |
| nhr-139 | 0.47 |
| F27D4.4 | 0.45 |
| ceh-82 | 0.44 |
| nhr-140 | 0.44 |
| nhr-104 | 0.44 |
| cep-1 | 0.43 |
| ztf-13 | 0.43 |
| hmg-5 | 0.42 |
| nhr-64 | 0.42 |
| sup-35 | 0.42 |
| nhr-246 | 0.42 |
| duxl-1 | 0.42 |
| nhr-90 | 0.42 |
| C01F6.9 | 0.41 |
| nhr-210 | 0.41 |
| nhr-112 | 0.40 |
| hlh-12 | 0.40 |
| ceh-7 | 0.40 |
| zip-7 | 0.39 |
| ccch-3 | 0.39 |
| hsf-1 | -0.58 |
| nhr-47 | -0.58 |
| spr-4 | -0.59 |
| madf-4 | -0.59 |
| nhr-36 | -0.61 |
| sem-4 | -0.61 |
| tag-97 | -0.61 |
| egl-13 | -0.61 |
| cey-1 | -0.62 |
| skn-1 | -0.62 |
| nhr-48 | -0.63 |
| nhr-138 | -0.63 |
| nhr-1 | -0.64 |
| nhr-26 | -0.64 |
| nhr-101 | -0.64 |
| daf-19 | -0.65 |
| ctbp-1 | -0.65 |
| daf-3 | -0.67 |
| ztf-9 | -0.67 |
| mbr-1 | -0.68 |
| hlh-10 | -0.68 |
| tag-68 | -0.71 |
| hif-1 | -0.71 |
| nhr-145 | -0.77 |
| camt-1 | -0.79 |

### ChIP peaks enriched

|  |  |  |  |  |
| --- | --- | --- | --- | --- |
| **Gene** | **Experiment** | **Number of upstream peaks** | **Enrichment** | **FDR corrected p** |
| lin-15 | LIN-15B\_Larvae-L4-stage | 6 | 9.87 | 0.0010 |
| aha-1 | AHA-1\_Fed-L1-stage-larvae | 5 | 12.82 | 0.0016 |
| ama-1 | AMA-1\_Larvae-L3-stage | 6 | 6.85 | 0.0064 |
| nfya-1 | NFYA-1\_Young-adult | 6 | 6.57 | 0.0079 |
| lin-35 | LIN-35\_Starved-L1-stage-larvae | 6 | 5.93 | 0.0130 |
| lin-35 | LIN-35\_Fed-L1-stage-larvae | 11 | 2.80 | 0.0170 |
| dpl-1 | DPL-1\_Young-adult | 10 | 3.07 | 0.0180 |
| lin-13 | LIN-13\_Larvae-L1-stage | 5 | 7.11 | 0.0190 |
| pha-4 | PHA-4\_Young-adult | 7 | 4.36 | 0.0220 |
| lsy-2 | LSY-2\_Larvae-L4-stage | 6 | 5.07 | 0.0270 |
| lsy-2 | LSY-2\_Larvae-L2-stage | 6 | 5.05 | 0.0280 |
| ceh-39 | CEH-39\_Embryos | 7 | 4.08 | 0.0320 |
| ham-1 | HAM-1\_Fed-L1-stage-larvae | 9 | 3.06 | 0.0370 |
| nhr-76 | NHR-76\_Larvae-L4-stage | 7 | 3.86 | 0.0430 |
| nhr-6 | NHR-6\_Larvae-L4-stage | 8 | 3.32 | 0.0460 |
| nhr-25 | NHR-25\_Larvae-L2-stage | 10 | 2.68 | 0.0470 |
